# How long does a knee replacement last? A systematic review and meta-analysis of case series and national registry reports with more than 15 years of follow-up

**DOI:** 10.1016/S0140-6736(18)32531-5

**Published:** 2019-02-16

**Authors:** Jonathan T Evans, Robert W Walker, Jonathan P Evans, Ashley W Blom, Adrian Sayers, Michael R Whitehouse

**Affiliations:** a Musculoskeletal Research Unit, Translational Health Sciences, Bristol Medical School, University of Bristol, Southmead Hospital, Bristol, UK; b Department of Trauma and Orthopaedics, Torbay and South Devon NHS Foundation Trust, Torquay, UK; c Health and Policy Research Group, University of Exeter, Exeter, UK; d National Institute for Health Research Bristol Biomedical Research Centre, University Hospitals, Bristol NHS Foundation Trust, University of Bristol, Bristol, UK

## Abstract

**Background:**

Knee replacements are the mainstay of treatment for end-stage osteoarthritis and are effective. Given time, all knee replacements will fail and knowing when this failure might happen is important. We aimed to establish how long a knee replacement lasts.

**Methods:**

In this systematic review and meta-analysis, we searched MEDLINE and Embase for case series and cohort studies published from database inception until July 21, 2018. Articles reporting 15 year or greater survival of primary total knee replacement (TKR), unicondylar knee replacement (UKR), and patellofemoral replacements in patients with osteoarthritis were included. Articles that reviewed specifically complex primary surgeries or revisions were excluded. Survival and implant data were extracted, with all-cause survival of the knee replacement construct being the primary outcome. We also reviewed national joint replacement registry reports and extracted the data to be analysed separately. In the meta-analysis, we weighted each series and calculated a pooled survival estimate for each data source at 15 years, 20 years, and 25 years, using a fixed-effects model. This study is registered with PROSPERO, number CRD42018105188.

**Findings:**

From 4363 references found by our initial search, we identified 33 case series in 30 eligible articles, which reported all-cause survival for 6490 TKRs (26 case series) and 742 UKRs (seven case series). No case series reporting on patellofemoral replacements met our inclusion criteria, and no case series reported 25 year survival for TKR. The estimated 25 year survival for UKR (based on one case series) was 72·0% (95% CI 58·0–95·0). Registries contributed 299 291 TKRs (47 series) and 7714 UKRs (five series). The pooled registry 25 year survival of TKRs (14 registries) was 82·3% (95% CI 81·3–83·2) and of UKRs (four registries) was 69·8% (67·6–72·1).

**Interpretation:**

Our pooled registry data, which we believe to be more accurate than the case series data, shows that approximately 82% of TKRs last 25 years and 70% of UKRs last 25 years. These findings will be of use to patients and health-care providers; further information is required to predict exactly how long specific knee replacements will last.

**Funding:**

The National Joint Registry for England, Wales, Northern Ireland, and Isle of Man and the Royal College of Surgeons of England.

## Introduction

Osteoarthritis of the knee is a common and potentially debilitating condition. The mainstay of treatment for end-stage disease is knee replacement, and this procedure has been shown to be effective in most cases.^1^ Depending upon the extent of disease, replacement surgery can take the form of total knee replacement (TKR), unicondylar knee replacement (UKR), or patellofemoral replacement (PFR). In TKR, all the articular surfaces of the tibiofemoral joint are replaced (with or without the articulating surface of the patella). UKR and PFR are suitable for disease confined to one compartment and only that compartment is replaced. TKR, UKR, and PFR are now some of the most common surgical procedures worldwide with a marked secular increase.

The aim of knee replacement surgery is the long-term relief of pain and restoration of function. Unfortunately, knee replacements fail for a variety of reasons, including loosening, infection, persistent pain, and instability,^2^ and might require revision within the lifetime of the recipient. Knowing what the long-term failure rates are is important to facilitate resource planning, medicolegal assessment, benchmarking of different implants, and the provision of informed consent to patients. Given enough time, all knee replacements will fail and need to be revised. Revision is expensive^3^, ^4^ and results in worse outcomes than primary surgery.

The National Institute of Health and Care Excellence set a UK benchmark in 2014, which stated that individual hip replacement components are only recommended if 5% or fewer need revision at 10 years,^5^ but no equivalent benchmark exists for knee replacement in either the medium (10 years) or long term.

In the UK, in 2016, the typical patient requiring knee replacement was aged 69 years with a body-mass index of 31 kg/m^2^. Almost all (99%) knee replacements were done in patients with osteoarthritis and 56% of patients were women.^3^ In 2016, 108 713 knee replacement procedures were done in England, Wales, Northern Ireland, and the Isle of Man.^3^ 60 different implants were used for primary TKR, comprising 90·1% of all knee replacement procedures; 18 different UKR implants and nine different PFR implants were used.^6^

**Research in context**

**Evidence before this study**

We searched MEDLINE and Embase for systematic reviews and meta-analyses published in English. Our search identified 34 systematic reviews, but none of these articles produced a combined survival estimate with more than 15 years follow-up, and although many reviews compared subgroups (eg, cemented *vs* cementless), we believe these studies to be prone to selection bias. Before the advent of national joint replacement registers, case series were the only sources of survival estimates for knee replacements. A previous analysis of the Finnish Arthroplasty Registry provided an estimate of the survival of total knee replacement at 15 years of 88·7% (95% CI 88·5–88·9) for total knee replacement (TKR) and 69·6% (68·2–70·9) for unicondylar knee replacement (UKR). An analysis that used the UK Clinical Practice Research Datalink (CPRD) database was published in 2017, and estimated survival of TKR to be 89·7% (95% CI 87·5–91·5) at 20 years.

**Added value of this study**

To our knowledge, we have provided the first simple and generalisable estimate of the survival of knee replacements at 25 years, providing an answer to the question—how long does a knee replacement last? Our findings showed that approximately 82% of TKRs and 70% of UKRs last for 25 years.

**Implications of all the available evidence**

Our findings, combined with those of previous registry analyses and the analysis of the CPRD data by Bayliss and colleagues, are of use to patients, people providing and commissioning health-care services, and those needing an estimate of knee replacement survival for medicolegal purposes.

In this study, we wish to answer a simple question that is posed to us by our patients, multiple times per day—how long does a knee replacement last?

## Methods

### Search strategy and selection criteria

This systematic review and meta-analysis followed a predefined protocol registered with PROSPERO (CRD42018105188) and adhered to PRISMA guidelines.^7^ We did one systematic review and meta-analysis of case series and cohort studies reporting survival outcomes of knee replacements and a second meta-analysis of national joint replacement registries with more than 15 years of follow-up.

We systematically searched MEDLINE and Embase for case series and cohort studies that reported survival outcomes of knee replacements, published between commencement of each database and July 21, 2018. The search was done through the Ovid SilverPlatter platform and contained keywords relating to knee replacement, survival, and medical subject heading terms (see appendix for details of exact search terms used). Bibliographies of all included articles, as well as review articles, were manually screened for additional citations (JTE). Nine studies were excluded because they were not written in English. Studies were included if they involved patients undergoing any type of knee replacement (TKR, UKR, or PFR) for osteoarthritis or a predominantly unselected patient group (eg, studies that investigated only one or two indications, such as rheumatoid arthritis, were excluded). Survival reports of specific implants with a mean or median follow-up of more than 15 years were required. Articles that reviewed specifically complex primary surgeries or revisions were excluded, because these types of procedures have different survivorship of the implants. Some articles reported survivorship from registry data; we excluded these studies because their inclusion would lead to duplication of data identified from the second data source. Systematic reviews were retrieved, and citations searched, but pooled data from the reviews themselves were not included because their inclusion would result in duplication.

The first national joint replacement registry was founded in Sweden in 1975; at the time of writing, six registries have more than 15 years of potential follow-up for knee replacement (those in Australia, Denmark, Finland, New Zealand, Norway, and Sweden). For the analysis of data from national joint replacement registries, we reviewed websites and the most recent annual reports of national joint replacement registries with 15 years of follow-up at the time of data collection (July 21, 2018) for 15 year or longer survival data on TKR, UKR, or PFR constructs. These national registries collect data on all patients undergoing knee replacements in both public and private hospitals and aim to include all joint replacements in their cohort.

### Abstract screening and data extraction

The abstracts of journal articles were screened by three reviewers (JTE, RWW, and JPE) using the web application Rayyan,^8^ and in cases of disagreement were included for full review. Data were extracted twice on publication date, implant type, fixation technique, number of knee replacements, age and sex of recipient, indication for knee replacement, loss to follow-up, and summary survivorship estimates (including CIs), when available. Data were not extracted from figures to prevent potential inaccuracy, particularly in the case of older, low-resolution figures. Any discrepancy between the twice-extracted data was rectified by review of the full text by all reviewers, after which there were no cases of disagreement.

### Data analysis

Our primary exposure was the make and model of the knee replacement construct and our primary outcome was all-cause revision of any part of this construct. We required estimates of survival for specific constructs because we believe that this make and model of the construct fundamental to the outcome of surgery. Aggregated data from multiple constructs would not allow this level of detail and would thus hide the variability in performance between constructs. Statistical analysis was done with Stata 15 (release 15). Knee replacement construct survival estimates at 15 years, 20 years, and 25 years, assuming survivorship approximated risk, were pooled with meta-analysis. Each series was weighted according to its standard error (calculated from published CIs). A fixed-effects model was used. Variability between studies and publication bias were assessed using visual representation of the data.

Data from registries were analysed in the same way as data from the case series to produce equivalent forest plots. The contribution each implant series made was weighted according to the standard error of that individual estimate.

Study quality was assessed using the non-summative four-point system (consecutive cases, multicentre, under 20% loss to follow-up, and using multivariable analysis) developed by Wylde and co-workers.^9^ This system was preferred over the summative MINORS score because of the high loss to follow-up in joint replacement case series and because some of the scoring criteria were not relevant to joint replacement.^10^

This study is registered with PROSPERO, number CRD42018105188.

### Role of the funding source

The funder of the study had no role in study design, data collection, data analysis, data interpretation, or writing of the report. The corresponding author had full access to all the data in the study and had final responsibility for the decision to submit for publication.

## Results

**Figure 1:**
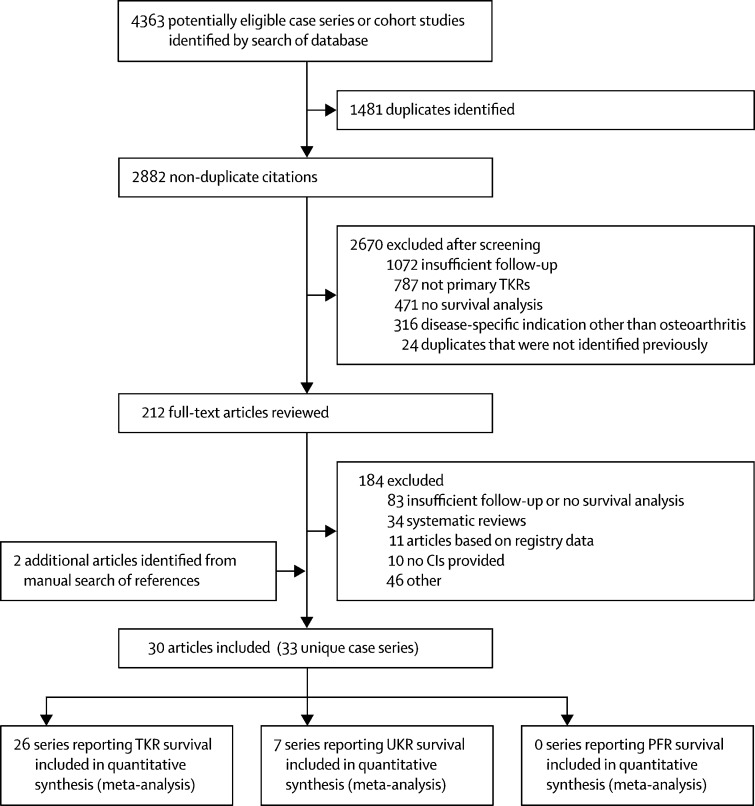
Flow chart of case series and cohort study selection TKR=total knee replacement. UKR=unicondylar knee replacement. PKR=patellofemoral replacement.

The search of published case series produced 4363 articles. 1481 duplicates were removed, leaving 2882 articles for screening (figure 1). After screening, 212 full-text articles remained for review, with two additional citations identified through manual search of references and reviews (appendix). Nine articles that were not written in English were excluded. Following review of full-text articles, 30 journal articles reporting 33 unique case series were included; these articles reported 7232 knee replacements (range 50–1000). 26 case series reported survival of TKRs and seven reported survival of UKRs; no case series reporting on PFR met our inclusion criteria. A full list of included articles and survival estimates can be found in the appendix. A summary of study-level and patient-level characteristics of each data source is provided (table 1).

**Table 1:** Study-level and participant-level characteristics of contributing data sources

	**Individual case series articles**		**Australian Orthopaedic Association National Joint Replacement Registry annual report, 2017**		**Finnish Arthroplasty Report, November, 2017**	
	TKR	UKR	TKR	UKR	TKR	UKR
**Study-level characteristics**						
Location	26 articles in ten countries	Seven articles in four countries	Australia	Australia	Finland	Finland
Number of unique implant series included	26	7	24	0	23	5
Year of publication	1999–2018	1999–2013	2017	NA	2017	2017
**Participant-level characteristics**						
Total joint replacements included	6490	742	209 435	0	89 856	7714
Mean age (years)	67·3*	68·7*	68·5†	NA	65–74‡§	65–74‡§
Proportion of female patients	55·4%¶	65·0%¶	56·8%†	NA	68·4%†§	68·4%†§
Proportion of arthroplasties implanted for osteoarthritis	87·5%‖	93·7%‖	97·6%†	NA	88·9%†§	88·9%†§

TKR=total knee replacement. UKR=unicondylar knee replacement. NA=not applicable.

* Weighted mean for age by number of arthroplasties in series.

† All primary knee operations in the report (not just those included in study).

‡ Exact value not reported, median within this age range.

§ Note that these values are the same in the TKR and UKR columns because the Finnish Arthroplasty Registry does not differentiate between TKR and UKR.

¶ Weighted proportion of female patients by number in study if the proportion of women was reported.

‖ Weighted proportion by number of arthroplasties in series if reported.

Quality assessment of the case series revealed that 22 (67%) of 33 were consecutive, 1 (3%) of 33 were multicentre, all 33 (100%) had less than 20% loss to follow-up, and only 4 (12%) of 33 included multivariable analyses. These proportions reflect the fact that, in general, the quality of published case series is low.

**Figure 2:**
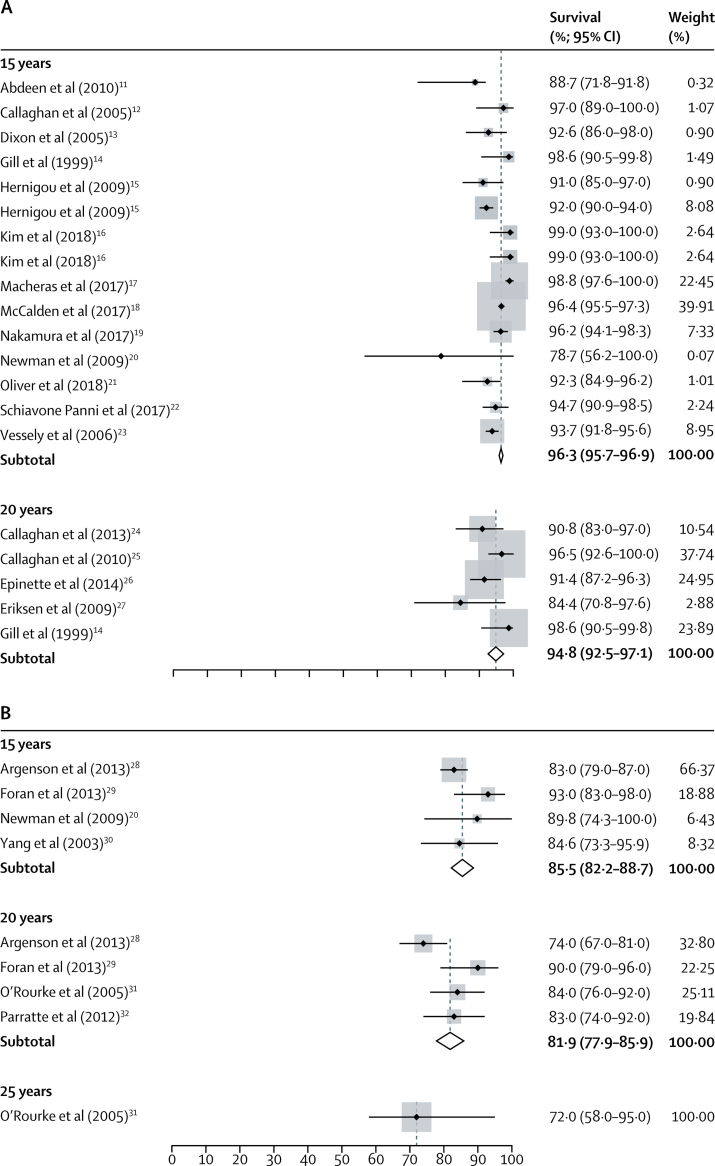
Forest plot of estimates for reported survival of knee replacements from case series^11^, ^12^, ^13^, ^14^, ^15^, ^16^, ^17^, ^18^, ^19^, ^20^, ^21^, ^22^, ^23^, ^24^, ^25^, ^26^, ^27^, ^28^, ^29^, ^30^, ^31^, ^32^ (A) Total knee replacements at 15 years and 20 years. (B) Unicondylar knee replacements at 15 years, 20 years, and 25 years. The CIs for individual point estimates are shown with horizontal lines. The surrounding box shows the contribution made by that individual estimate to the overall pooled estimate, weighted by the standard error of that individual series.

26 unique case series reported survival for 6490 TKRs, with follow-up ranging from 15 years to 23 years. 14 series reported survival at 15 years (4137 TKRs) and five at 20 years (763 TKRs). Not all series reported survival at exactly 15 years or 20 years and some series reported survival at more than one timepoint. Pooled analysis of data derived from case series reported at exactly 15 years or 20 years showed an all-cause construct survivorship of 96·3% (95% CI 95·7–96·9) at 15 years and 94·8% (92·5–97·1) at 20 years. Figure 2A shows a forest plot for the meta-analysis of data on TKRs derived from case series at 15 years and 20 years.

Pooled survival at each timepoint is shown in table 2. When we rounded survival at timepoints that were not exactly 15 years or 20 years down to the closest timepoint (to include as many case series as possible in our analyses), pooled survival was 96·3% (95% CI 95·7–96·8) at 15 years and 92·0% (90·1–93·8) at 20 years (appendix).

**Table 2:** Pooled estimates of survival for each available timepoint

	**Number of series**	**Total number of arthroplasties at the start of the series**	**Pooled survival estimate (95% CI)**
**Total knee replacement**			
15 years	15	4137	96·3% (95·7–96·9)
16·8 years	2	228	96·3% (93·5–99·0)
18 years	2	572	93·8% (81·0–96·0)
19 years	2	356	96·1% (92·8–99·4)
20 years	5	763	94·8% (92·5–97·1)
20·8 years	1	160	86·9% (80·4–91·5)
22 years	1	163	82·1% (76·2–88·0)
23 years	1	489	89·0% (82·0–93·0)
**Unicondylar knee replacement**			
15 years	4	387	85·5% (82·2–88·7)
16 years	1	113	81·3% (67·7–94·8)
20 years	4	437	81·9% (77·8–85·9)
22 years	1	140	84·0% (75·0–93·0)
25 years	1	136	72·0% (58·0–95·0)

Seven unique case series reported survival for 742 UKRs, with follow-up ranging from 15 years to 25 years. Four series reported survival at 15 years (387 UKRs), four at 20 years (437 UKRs), and one at 25 years (136 UKRs). Not all series reported survival at exactly 15 years, 20 years, or 25 years, and some series reported survival at more than one timepoint. Pooled analysis of data derived from case series reported at exactly 15 years, 20 years, and 25 years showed an all-cause construct survivorship of 85·5% (95% CI 82·2–88·7) at 15 years, 81·9% (77·9–85·9) at 20 years, and 72·0% (58·0–95·0) at 25 years. Figure 2B shows a forest plot for the meta-analysis of the data on UKRs derived from case series at 15 years, 20 years, and 25 years.

Pooled survival at each timepoint can be seen in table 2. When we rounded survival at timepoints that were not exactly 15 years, 20 years, or 25 years down to the closest timepoint (to include as many series as possible in our analyses), pooled survival was 85·3% (95% CI 82·0–88·6) at 15 years, 82·2% (78·6–85·9) at 20 years, and 72·0% (58·0–95·0) at 25 years (appendix).

The search of joint replacement registry reports yielded 47 series reporting TKRs and five reporting UKRs. The estimates from these series all originated from the Australian and Finnish registries. The other four arthroplasty registries with 15 years of potential follow-up did not provide survival estimates that were broken down by implant type, and therefore we could not use them in our analyses.

All 47 TKR series reported survival analyses at 15 years (299 291 TKRs), 20 series reported survival at 20 years (88 532 TKRs), and 14 series at 25 years (76 651 TKRs). The pooled survival data derived from registry data showed an all-cause construct survivorship of 93·0% (95% CI 92·8–93·1) at 15 years, 90·1% (89·7–90·4) at 20 years, and 82·3% (81·3–83·2) at 25 years. We obtained 15 year estimates from both the Australian and the Finnish registries and the 20 year and 25 year estimates were exclusively from the Finnish registry.

We obtained all data on UKRs exclusively from the Finnish registry. All five UKR series reported survival analyses at 15 years (7714 UKRs), four series reported survival at 20 years (3935 UKRs), and four series at 25 years (3935 UKRs). The pooled survival data derived from registry data showed an all-cause construct survivorship of 76·5% (95% CI 75·2–77·7) at 15 years, 71·6% (69·6–73·6) at 20 years, and 69·8% (67·6–72·1) at 25 years.

**Figure 3:**
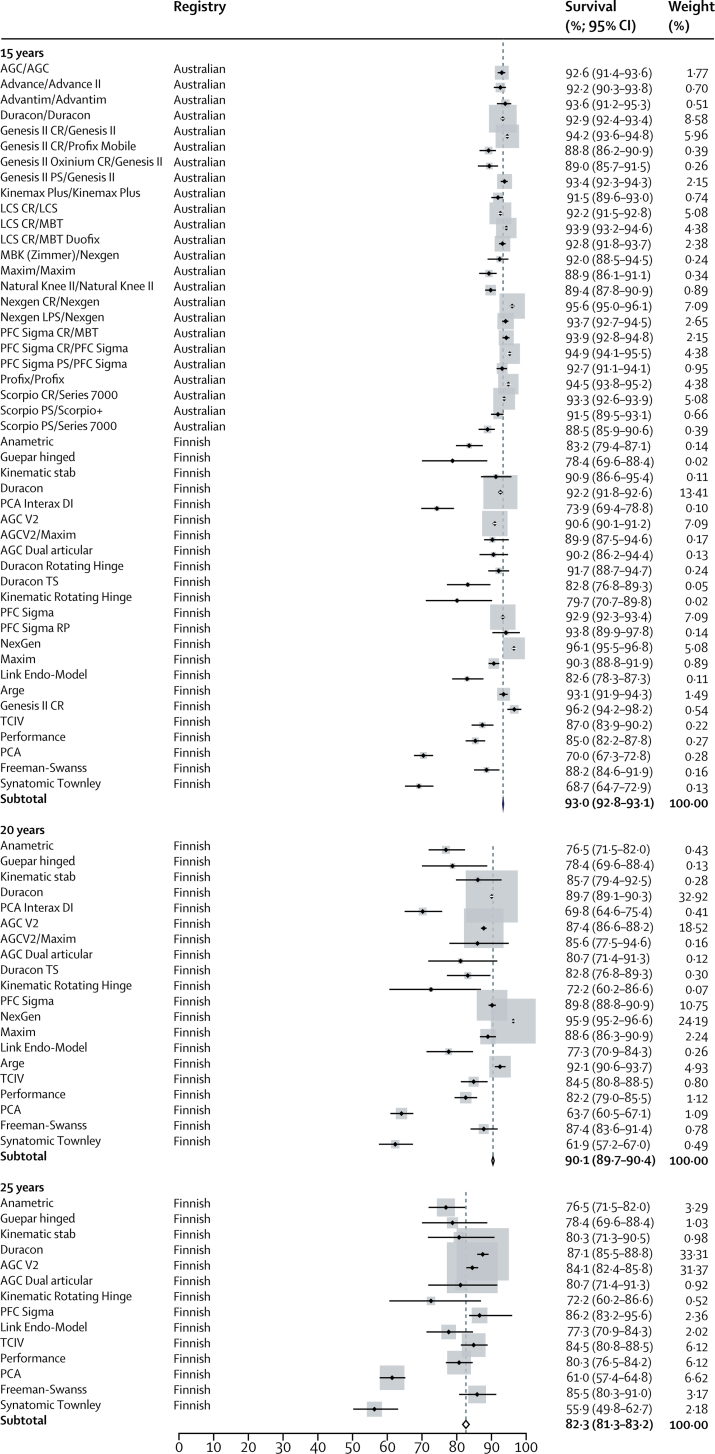
Forest plot of estimates for reported survival of total knee replacements from registry reports at 15 years, 20 years, and 25 years

**Figure 4:**
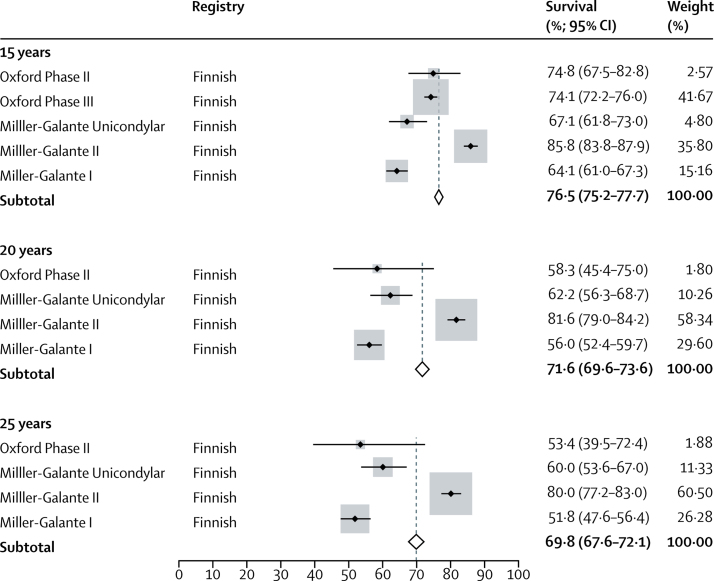
Forest plot of estimates for reported survival of unicondylar knee replacements from registry reports at 15 years, 20 years, and 25 years

Forest plots for the meta-analysis of data derived from joint replacement registry reports for TKR and UKR are provided in Figure 3, Figure 4.

**Figure 5:**
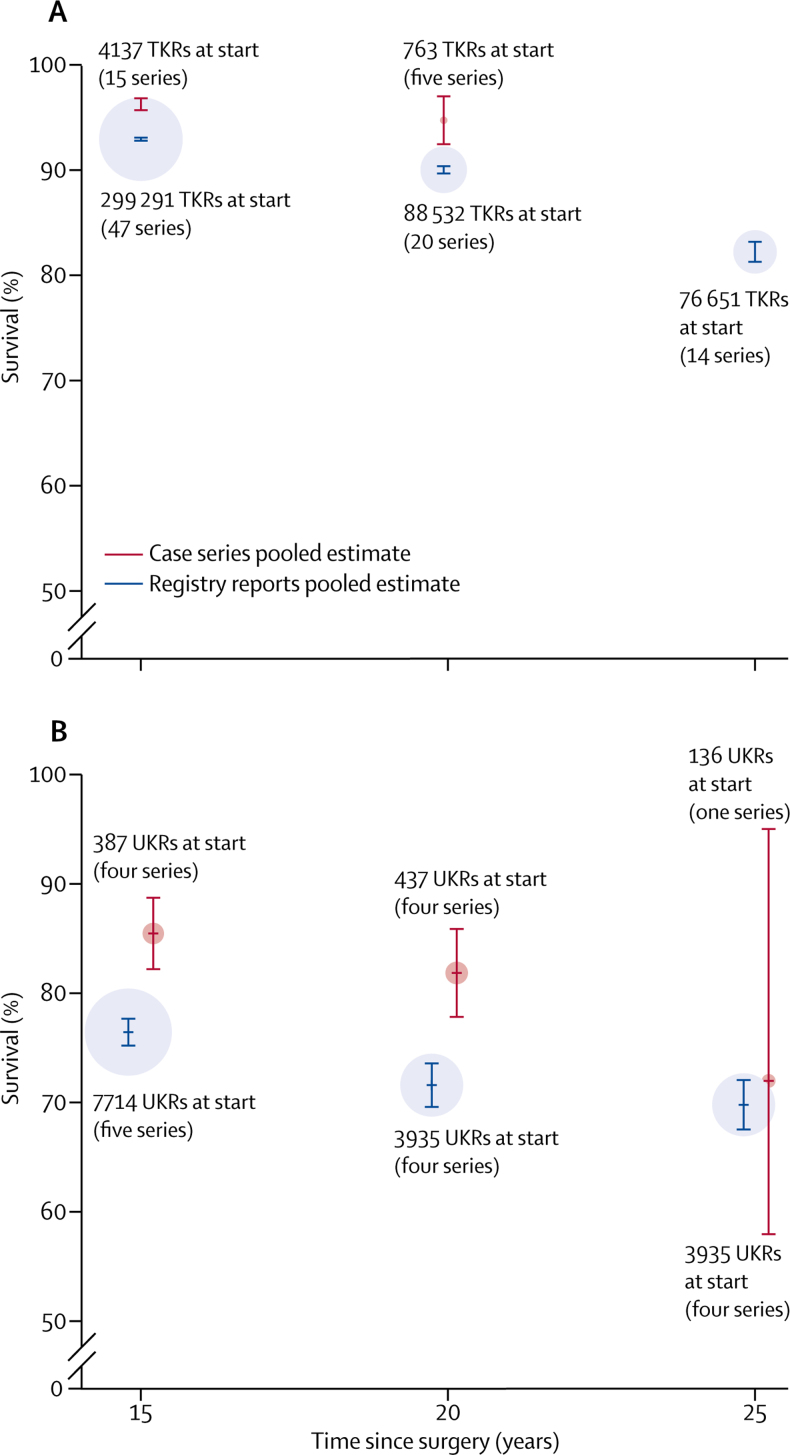
Comparison of pooled survival estimates for knee replacements from case series and registry reports at 15 years, 20 years, and 25 years (A) Total knee replacements. (B) Unicondylar knee replacements. The size of the circle representing each point estimate is proportional to the total number of hip replacements at the start of all the series contributing to that pooled estimate. Bars indicate 95% CIs. TKR=total knee replacement. UKR=unicondylar knee replacements.

A comparison of the point estimates at each timepoint for the two sources is shown for TKR and UKR (figure 5).

## Discussion

The question of how long does a knee replacement last is frequently asked by patients, and to date, we have not had a generalisable answer. The implant itself is fundamental to the survival outcome of surgery and each individual series should be considered as a different patient cohort. We have used individual estimates for each implant to synthesise a single pooled estimate, weighting the estimates according to standard error. This type of analysis, which derives an overall estimate according to how frequently each implant has been used, is unique to our study. The pooled registry data presented here show that 82·3% of TKRs and 69·8% of UKRs last 25 years. Only one case series reported the 25 year survival of UKRs (72·0%, 95% CI 58·0–95·0) and no TKR series provided 25 year results. Case series at 20 years of follow-up suggest better survival of TKR and UKR than comparable registry data. Although survival of a knee replacement prosthesis is important, it is not the only measure of success. One in five patients who undergo TKR for osteoarthritis reports an unfavourable pain outcome after surgery,^33^ and given our results, not all these patients seem to undergo revision surgery. The age of the patients in our study is similar to that reported by the largest national registries, such as the National Joint Registry for England, Wales, Northern Ireland, and the Isle of Man (NJR) and the Swedish Knee Arthroplasty Register (SKAR), suggesting that our data are likely to be generalisable. The proportion of female patients appears to be higher in the Finnish Arthroplasty Register than in the Australian registry or the pooled results of the case series, and this high proportion might affect the generalisability of the results. Given the secular changes in the sex distribution of patients receiving knee replacements seen in the SKAR, we expect this effect to be small.^34^ In the NJR, the median age of patients undergoing knee replacement surgery is 70 years and 57% are women, and similarly the SKAR reports the mean age of patients having knee replacements in 2016 to be 69 years and 57% of patients to be women.^2^, ^34^

The higher survival estimates at 20 years reported by case series are notable, but not surprising. Similar findings were seen by Pabinger and colleagues,^35^ who reported the mean proportion of TKRs needing revision to be 50% higher in registries than in case series. We also saw the same effect in our analysis of the survival of hip replacements.^36^ Differences in reported survival between registries and case series could be due to bias inherent to the reporting of case series, including selection and reporting bias. Our results support this theory by showing publication bias in case series. Several registry TKR construct series report survival that is well below our pooled estimate (figure 3); however, this effect is not present in case series (figure 2A). These findings suggest that the most poorly performing constructs are not reported and published in case series.

We identified 34 systematic reviews in our search, many of which attempted to report pooled survival of knee replacement. However, almost all these reviews focus on the comparison of different types of knee replacement (cemented *vs* cementless, mobile *vs* fixed bearing, or UKR *vs* TKR); we believe that, methodologically, these reviews are susceptible to selection bias, and we therefore created one pooled result for each type of knee replacement. In 2017, van der List and colleagues^37^ published a review that gave a 15 year survival estimate extrapolated from shorter-term data and only included cementless implants, and so is susceptible to selection bias. Only 15·1% of all primary knee operations in the 2016 NJR were reported to use cementless implants, and so their results are not generalisable.^3^ A study in 2014 by Niinimaki and co-workers^38^ analysed Finnish registry data to estimate the age and sex-adjusted survival of TKR and UKR at 15 years. The authors provide 15 year survival estimates of 88·7% (95% CI 88·5–88·9) for TKR and 69·6% (68·2–70·9) for UKR, figures that are lower than the estimates produced by our pooled analyses. These findings might be because Niinimaki and co-workers^38^ only used data from 1985 to 2011, and did not include results from the Australian registry. The authors noted the varying survival between different implants, further supporting our differentiation by the implant used. Analysis of the UK Clinical Practice Research Datalink database by Bayliss and colleagues^39^ estimated survival of total knee replacement to be 89·7% (95% CI 87·5–91·5) at 20 years, which is consistent with our analysis.

We excluded ten articles that did not report CIs and seven that did not report all-cause construct survival. These articles could have increased the number of series included in our study by 50%, which further highlights the heterogeneity and low-quality reporting of case series. The methods of survival analysis varied among identified articles, with many using Kaplan-Meier;^40^ however, life-table methods, such as those described by Armitage,^41^ were also used.^11^ Some articles reported 15 year survival of implants before 15 years mean follow-up had been reached, and therefore did not meet our inclusion criteria. We noted a trend towards the use of a competing risks method, believed by some to be a more accurate estimate of survival, given the high mortality seen in arthroplasty cohorts.^42^, ^43^, ^44^, ^45^ As we discuss in another paper,^46^ competing risks and Kaplan-Meier methods are not more or less accurate than each other, but simply report different phenomena. The heterogeneity observed in case series reinforces the importance of results from arthroplasty registries that are more consistent in their use of analysis techniques.

Our study did have some limitations. Our pooled data were not adjusted or stratified by patient factors that might have a role in determining survivorship, such as age, sex, or indication for the primary procedure. This detail is not provided in the data available to us and would require collaboration between registries with individual patient data. We provide an aggregated estimate for survival in all patients and, to our knowledge, this report is the first of its type with this length of follow-up. As with all survival reports, we cannot account for a surgeon's willingness to revise a knee replacement based upon individual patient factors. This revision threshold might change between countries and affect the generalisability of results between nations. Our pooled registry results are drawn only from Australia and Finland, with 20 year and 25 year TKR data and all UKR data coming exclusively from Finland. Although this small number of countries provides limited geographical capture, the number of knee replacements included from registries is still far greater than that from case series; for TKR this number is 299 291 compared with 6490 knees, and in UKR this number is 7714 compared with 742 knees. We excluded papers that were not written in English, which removed nine further series. If all these case series had met the inclusion criteria, they might have altered our pooled results, but we expect this effect would have been small. We assume that survival estimates are equivalent to risks, for generating pooled estimates, and although the assumption that no censoring occurs (patients dying with a knee replacement in situ) is clearly violated, this assumption provides a useful method of aggregation in the absence of individual patient-level data. The number of UKRs at risk in the 25 year follow-up group was less than 25 constructs in two of our included registry series. Although the accuracy of Kaplan-Meier estimates should be questioned when the number at risk is this small, this sample size is reflected in the weighting of estimates for our pooled results. The strengths of our study include an inclusive and comprehensive design, a-priori inclusion criteria, and a realistic interpretation of survivorship accounting for all revision operations and not a limited or biased subset of a particular indication for revision. From the patient perspective, any subsequent re-operation carries risks, and therefore all revisions should be counted.

In conclusion, pooled survival derived from case series appears to show a more optimistic estimate than pooled registry data. Given this finding and the bias inherent in published case series, we believe registry data to be the more accurate estimate. Not enough information is yet available to tell us exactly how long a TKR or UKR lasts; however, using available arthroplasty registry data, 82% of TKRs and 70% of UKRs last 25 years in patients with osteoarthritis (video).

## Supplementary Materials

Supplementary appendix

Supplementary viceo A summary of the findings from two systematic reviews and meta-analyses of national arthroplasty registry reports.
