# Department of Error

**DOI:** 10.1016/S0140-6736(19)30415-5

**Published:** 2019-03-02

*Evans JT, Walker RW, Evans JP, Blom AW, Sayers A, Whitehouse MR. How long does a knee replacement last? A systematic review and meta-analysis of case series and national registry reports with more than 15 years of follow-up.* Lancet *2019; **393**: 655–63*—In this Article, the Summary has been corrected to include the National Institute for Health Research as a funder. This is an Open Access article under the CC BY 4.0 licence, and the copyright statement in the Summary has been updated accordingly. These corrections have been made to the online version as of Feb 20, 2019.

