# Supplementary material for: How long does a knee replacement last? A systematic review and meta-analysis of case series and national registry reports with more than 15 years of follow-up
Source: Lancet. 2019 Feb 16;393(10172):655–63. doi: 10.1016/S0140-6736(18)32531-5 (PMC6381229; doi:10.1016/S0140-6736(18)32531-5)

THE LANCET

Supplementary appendix

This appendix formed part of the original submission and has been peer reviewed. We post it as supplied by the authors.

Supplement to: Evans JT, Walker RW, Evans JP, Blom AW, Sayers A, Whitehouse MR. How long does a knee replacement last? A systematic review and meta-analysis of case series and national registry reports with more than 15 years of follow-up. *Lancet* 2019; **393:** 655–63.

Web Extra Material

Details of search strategy

Table 1: Search strategy formatted for search of Medline on Ovid Silver Platter

**Knee replacement**
Knee Prosthesis/ OR Arthroplasty, Replacement, Knee/ OR
(knee adj2 arthroplast$.mp) OR (knee adj2 replacement?.mp) OR
TKA.mp OR TKR.mp OR (TJR$.mp AND knee$.mp)
AND
**Survival**
Prosthesis Failure/ OR Survival Analysis/ OR
cox.mp OR proportional?hazard?.mp OR proportional hazard?.mp OR
cumulative?incidence?function.mp OR cumulative incidence function.mp OR CIF.mp OR
failure.mp OR
survival.mp OR survivor?ship.mp OR
revision?.mp OR
re?operation.mp OR re operation.mp OR
Kaplan?meier.mp OR Kaplan meier.mp OR KM.mp OR
product?limit?method.mp OR product limit method.mp
AND
**Case-series**
exp Cohort Studies/ OR
follow?up.mp OR follow-up.mp OR series.mp OR cohort.mp OR
registry.mp OR registries.mp
AND
**Long-term (minimum 15 years)**
long?term.mp OR long term.mp OR
(1#* adj1 year?.mp) OR (2#* adj1 year?.mp) OR (3#* adj1 year?.mp) OR (4#* adj1 year?.mp)
Survival Adjacent to Long term (within 20 words) AND Knee replacement AND Case-series

Reasons for exclusion

Table 2: Reasons for exclusion at screening

Primary reason for exclusion	Number
Insufficient follow-up	1072
Not an article regarding primary TKR<br>e.g. revision/osteotomy	787
No survival analysis	471
Disease specific cohort other than osteoarthritis	316
Duplicate not identified previously	24
**Total**	**2670**

Table 3: Reasons for exclusion at review of full text

Reason for exclusion	Number
Insufficient follow-up/no survival analysis	83
Systematic review	34
Article based on registry data	11
No confidence intervals provided	10
Article not in English language	9
Not a single implant	7
No all cause revision estimate	7
No survival estimate provided	6
Disease specific cohort other than osteoarthritis	5
Conference abstract – insufficient information	3
Not a case-series	3
No details on mean or minimum follow-up	2
Article withdrawn	1
Not primary knee replacement	1
Editorial on another article	1
Estimate outside confidence interval	1
**Total**	**184**

Articles contributing to meta-analysis

Table 4: Articles contributing to meta-analysis

Author	Year of publication	Implant type	Time point (years post op)	Survival Estimate (%)	Lower 95% confidence interval	Upper 95% confidence interval
Abdeen, A. R.[1]	2010	TKR	15·0	88·7	71·8	91·8
Abdeen, A. R.[1]	2010	TKR	19·0	88·7	71·8	91·8
Callaghan, J. J.[2]	2013	TKR	20·0	90·8	83·0	97·0
Callaghan, J. J.[3]	2005	TKR	15·0	97·0	89·0	100·0
Callaghan, J. J.[4]	2010	TKR	20·0	96·5	92·6	100·0
Dixon, M. C.[5]	2005	TKR	15·0	92·6	86·0	98·0
Epinette, J.[6]	2014	TKR	20·0	91·4	87·2	96·3
Eriksen, J.[7]	2009	TKR	20·0	84·4	70·8	97·6
Gill, G. S.[8]	1999	TKR	15·0	98·6	90·5	99·8
Gill, G. S.[8]	1999	TKR	20·0	98·6	90·5	99·8
Hernigou, P.[9]	2009	TKR	15·0	91·0	85·0	97·0
Hernigou, P.[9]	2009	TKR	15·0	92·0	90·0	94·0
Jordan, L. R.[10]	2005	TKR	19·0	97·0	93·0	100·0
Kim, Y.[11]	2012	TKR	16·8	95·0	91·0	100·0
Kim, Y.[11]	2012	TKR	16·8	97·0	93·0	100·0
Kim, Y.[12]	2018	TKR	15·0	99·0	93·0	100·0
Kim, Y.[12]	2018	TKR	15·0	99·0	93·0	100·0
Macheras, G. A.[13]	2017	TKR	15·0	98·8	97·6	100·0
Malin, A. S.[14]	2010	TKR	18·0	91·0	81·0	96·0
McCalden, R. W.[15]	2017	TKR	15·0	96·4	95·5	97·3
Melton, J. T. K.[16]	2012	TKR	18·0	94·5	88·2	95·5
Nakamura, S.[17]	2017	TKR	15·0	96·2	94·1	98·3
Newman, J.[18]	2009	TKR	15·0	78·7	56·2	100·0
Oliver, W. M.[19]	2018	TKR	15·0	92·3	84·9	96·2
Patil, S.[20]	2015	TKR	20·8	86·9	80·4	91·5
Pritchett, J. W.[21]	2015	TKR	23·0	89·0	82·0	93·0
Sabouret, P.[22]	2013	TKR	22·0	82·1	76·2	88·0
Schiavone Panni, A.[23]	2017	TKR	15·0	94·7	90·9	98·5
Vessely, M. B.[24]	2006	TKR	15·0	93·7	91·8	95·6
Argenson, J. A.[25]	2013	UKR	15·0	83·0	79·0	87·0
Argenson, J. A.[25]	2013	UKR	20·0	74·0	67·0	81·0
Foran, Jared R. H.[26]	2013	UKR	15·0	93·0	83·0	98·0
Foran, Jared R. H.[26]	2013	UKR	20·0	90·0	79·0	96·0
Newman, J.[18]	2009	UKR	15·0	89·8	74·3	100·0
O'Rourke, M. R.[27]	2005	UKR	20·0	84·0	76·0	92·0
O'Rourke, M. R.[27]	2005	UKR	25·0	72·0	58·0	95·0
Parratte, S.[28]	2012	UKR	20·0	83·0	74·0	92·0
Squire, M. W.[29]	1999	UKR	22·0	84·0	75·0	93·0
Yang, S.[30]	2003	UKR	15·0	84·6	73·3	95·9
Yang, S.[30]	2003	UKR	16·0	81·3	67·7	94·8

References of included articles

1. Abdeen AR, Collen SR, Vince KG. Fifteen-year to 19-year follow-up of the Insall-Burstein-1 total knee arthroplasty. *J Arthroplasty* 2010; **25**(2): 173-8.
2. Callaghan JJ, Beckert MW, Hennessy DW, Goetz DD, Kelley SS. Durability of a Cruciate-retaining TKA with modular tibial trays at 20 years knee. *Clin Orthop Relat Res* 2013; **471**(1): 109-17.
3. Callaghan JJ, O'Rourke MR, Iossi MF, et al. Cemented rotating-platform total knee replacement. a concise follow-up, at a minimum of fifteen years, of a previous report. *J Bone Joint Surg Am* 2005; **87**(9): 1995-8.
4. Callaghan JJ, Wells CW, Liu SS, Goetz DD, Johnston RC. Cemented rotating-platform total knee replacement: a concise follow-up, at a minimum of twenty years, of a previous report. *J Bone Joint Surg Am* 2010; **92**(7): 1635-9.
5. Dixon MC, Brown RR, Parsch D, Scott RD. Modular fixed-bearing total knee arthroplasty with retention of the posterior cruciate ligament. A study of patients followed for a minimum of fifteen years. *J Bone Joint Surg Am* 2005; **87**(3): 598-603.
6. Epinette J-A. Long lasting outcome of hydroxyapatite-coated implants in primary knee arthroplasty: a continuous series of two hundred and seventy total knee arthroplasties at fifteen to twenty two years of clinical follow-up. *Int Orthop* 2014; **38**(2): 305-11.
7. Eriksen J, Christensen J, Solgaard S, Schroder H. The cementless AGC 2000 knee prosthesis: 20-year results in a consecutive series. *Acta Orthop Belg* 2009; **75**(2): 225-33.
8. Gill GS, Joshi AB, Mills DM. Total condylar knee arthroplasty. 16- to 21-year results. *Clin Orthop Relat Res* 1999; (367): 210-5.
9. Hernigou P, Manicom O, Flouzat-Lachaniete CH, et al. Fifteen year outcome of the ceraver hermes posterior-stabilized total knee arthroplasty: safety of the procedure with experienced and inexperienced surgeons. *Open Orthop J* 2009; **3**: 36-9.
10. Jordan LR, Sorrells RB, Jordan LC, Olivo JL. The long-term results of a metal-backed mobile bearing patella. *Clin Orthop Relat Res* 2005; (436): 111-8.
11. Kim Y-H, Kim J-S, Choe J-W, Kim H-J. Long-term comparison of fixed-bearing and mobile-bearing total knee replacements in patients younger than fifty-one years of age with osteoarthritis. *J Bone Joint Surg Am* 2012; **94**(10): 866-73.
12. Kim Y-H, Park J-W, Kim J-S. 2017 Chitranjan S. Ranawat Award: Does Computer Navigation in Knee Arthroplasty Improve Functional Outcomes in Young Patients? A Randomized Study. *Clin Orthop Relat Res* 2018; **476**(1): 6-15.
13. Macheras GA, Galanakos SP, Lepetsos P, Anastasopoulos PP, Papadakis SA. A long term clinical outcome of the Medial Pivot Knee Arthroplasty System. *Knee* 2017; **24**(2): 447-53.

14. Malin AS, Callaghan JJ, Bozic KJ, et al. Routine surveillance of modular PFC TKA shows increasing failures after 10 years. *Clin Orthop Relat Res* 2010; **468**(9): 2469-76.

15. McCalden RW, Hart GP, MacDonald SJ, Naudie DD, Howard JH, Bourne RB. Clinical Results and Survivorship of the GENESIS II Total Knee Arthroplasty at a Minimum of 15 Years. *J Arthroplasty* 2017; **32**(7): 2161-6.

16. Melton JTK, Mayahi R, Baxter SE, Facek M, Glezos C. Long-term outcome in an uncemented, hydroxyapatite-coated total knee replacement: a 15- to 18-year survivorship analysis. *J Bone Joint Surg Br* 2012; **94**(8): 1067-70.

17. Nakamura S, Ito H, Nakamura K, Kuriyama S, Furu M, Matsuda S. Long-Term Durability of Ceramic Tri-Condylar Knee Implants: A Minimum 15-Year Follow-Up. *J Arthroplasty* 2017; **32**(6): 1874-9.

18. Newman J, Pydisetty RV, Ackroyd C. Unicompartmental or total knee replacement: the 15-year results of a prospective randomised controlled trial. *J Bone Joint Surg Br* 2009; **91**(1): 52-7.

19. Oliver WM, Arthur CHC, Wood AM, Clayton RAE, Brenkel IJ, Walmsley P. Excellent Survival and Good Outcomes at 15 Years Using the Press-Fit Condylar Sigma Total Knee Arthroplasty. *J Arthroplasty* 2018; **33**(8) 2524-2529

20. Patil S, McCauley JC, Pulido P, Colwell CW. How Do Knee Implants Perform Past the Second Decade? Nineteen- to 25-year Followup of the Press-fit Condylar Design TKA. *Clin Orthop Relat Res* 2015; **473**(1): 135-40.

21. Pritchett JW. Bicruciate-retaining Total Knee Replacement Provides Satisfactory Function and Implant Survivorship at 23 Years. *Clin Orthop Relat Res* 2015; **473**(7): 2327-33.

22. Sabouret P, Lavoie F, Cloutier JM. Total knee replacement with retention of both cruciate ligaments: a 22-year follow-up study. *Bone Joint J* 2013; **95**(7): 917-22.

23. Schiavone Panni A, Falez F, D'Apolito R, Corona K, Perisano C, Vasso M. Long-term follow-up of a non-randomised prospective cohort of one hundred and ninety two total knee arthroplasties using the NexGen implant. *Int Orthop* 2017; **41**(6): 1155-62.

24. Vessely MB, Whaley AL, Harmsen WS, Schleck CD, Berry DJ. The Chitranjan Ranawat Award: Long-term survivorship and failure modes of 1000 cemented condylar total knee arthroplasties. *Clin Orthop Relat Res* 2006; **452**: 28-34.

25. Argenson J-NA, Blanc G, Aubaniac J-M, Parratte S. Modern unicompartmental knee arthroplasty with cement: a concise follow-up, at a mean of twenty years, of a previous report. *J Bone Joint Surg Am* 2013; **95**(10): 905-9.

26. Foran JRH, Brown NM, Della Valle CJ, Berger RA, Galante JO. Long-term survivorship and failure modes of unicompartmental knee arthroplasty. *Clin Orthop Relat Res* 2013; **471**(1): 102-8.

27. O'Rourke MR, Gardner JJ, Callaghan JJ, et al. Unicompartmental Knee replacement: A minimum twenty-one-year followup, end-result study. *Clin Orthop Relat Res* 2005; (440): 27-37.

28. Parratte S, Pauly V, Aubaniac J-M, Argenson J-NA. No long-term difference between fixed and mobile medial unicompartmental arthroplasty. *Clin Orthop Relat Res* 2012; **470**(1): 61-8.

29. Squire MW, Callaghan JJ, Goetz DD, Sullivan PM, Johnston RC. Unicompartmental knee replacement. A minimum 15 year followup study. *Clin Orthop Relat Res* 1999; (367): 61-72.

30. Yang S, Hadlow S. Unicompartmental knee arthroplasty: is it durable? *N Z Med J* 2003; **116**(1183): U627.

Forest plots

Figure 1: Forest plot of estimates for reported survival for total knee replacements from case-series with reporting time rounded down to nearest 5 year interval

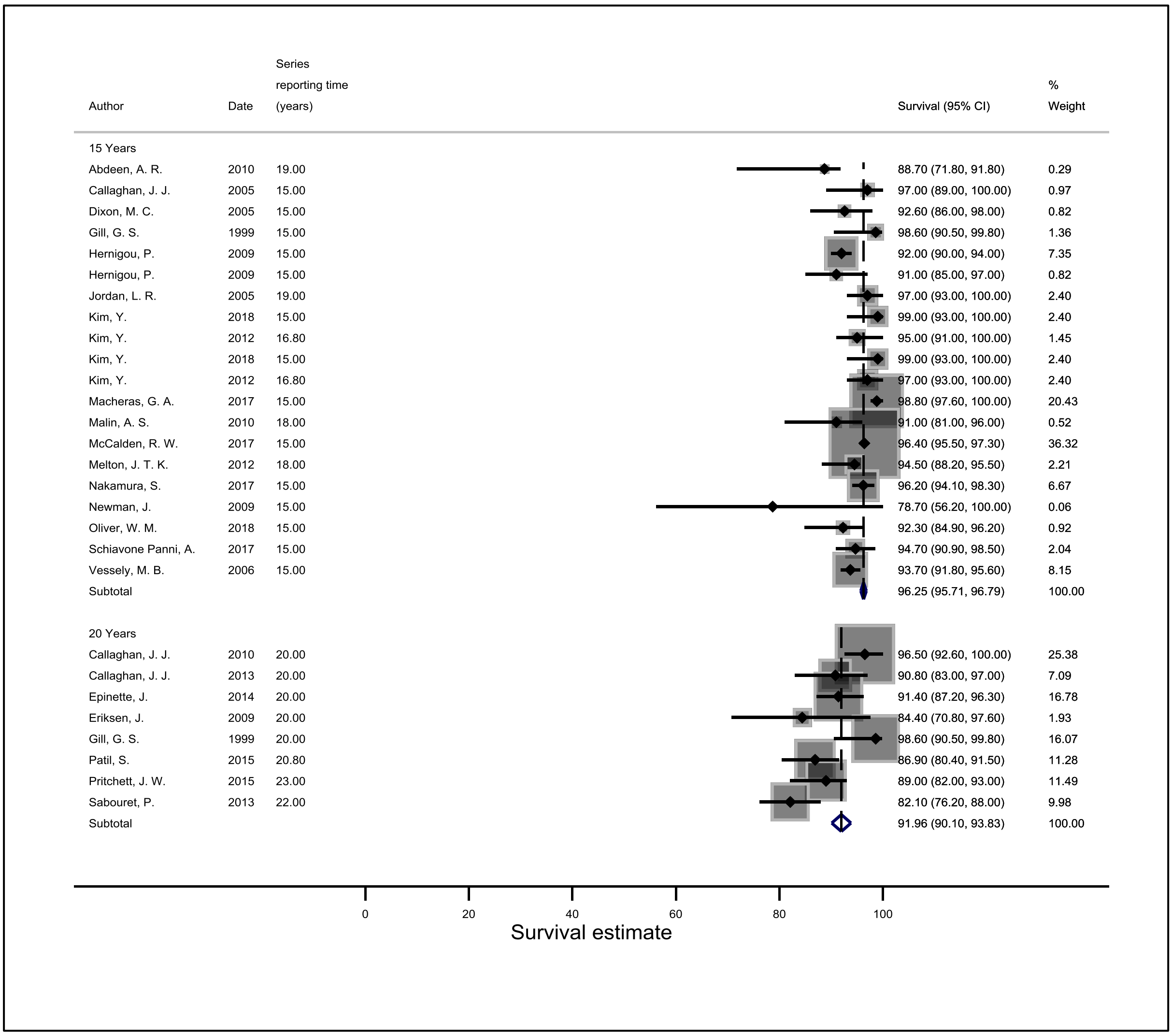

Figure 2: Forest plot of estimates for reported survival for unicondylar knee replacements from case-series with reporting time rounded down to nearest 5 year interval

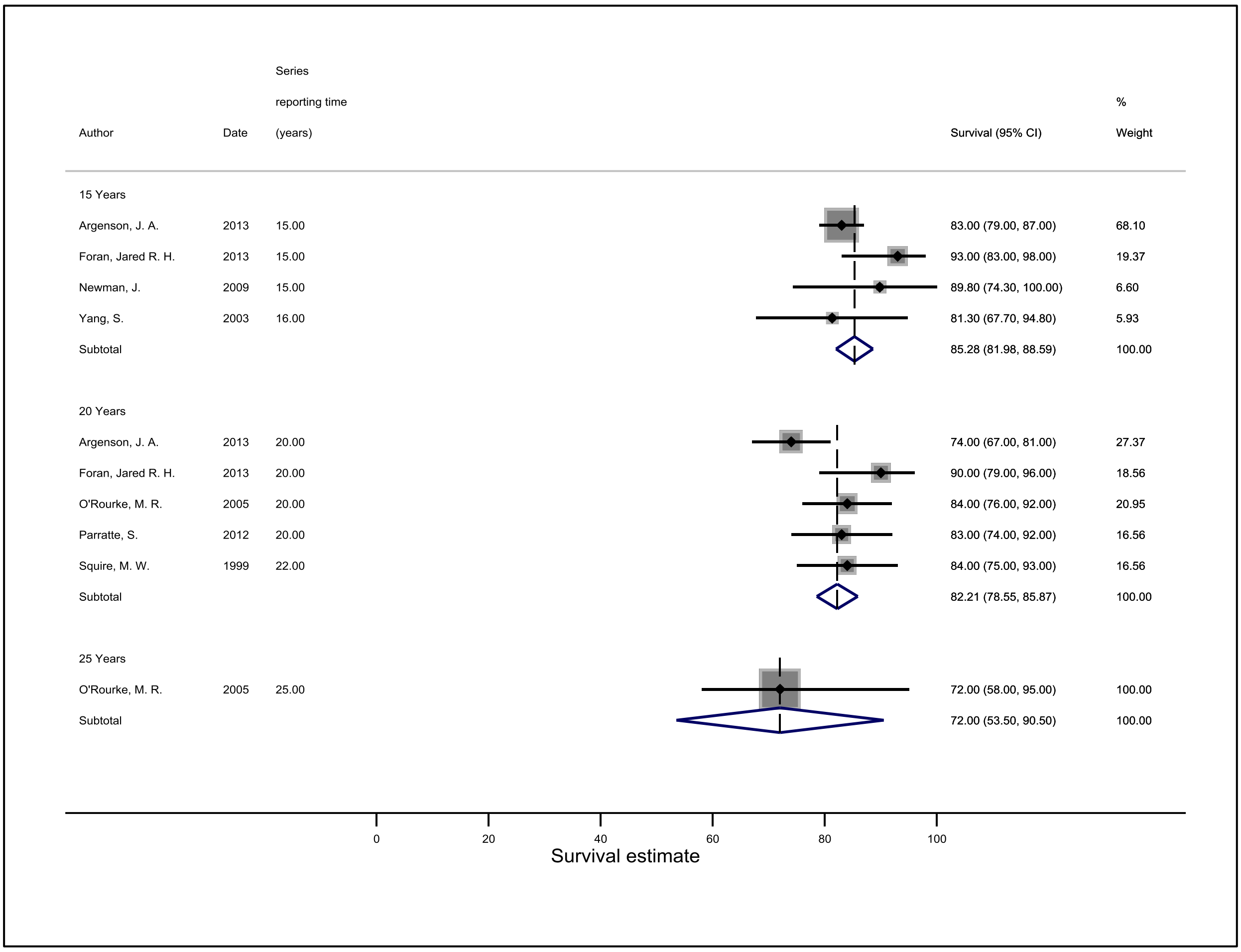

Supplement: Supplementary appendix [file mmc1.pdf]
